# Unveiling potential genetic markers: associations of *IGF1R* eon 13 and Intronic polymorphisms with growth traits in yak

**DOI:** 10.5194/aab-69-553-2026

**Published:** 2026-10-06

**Authors:** Xinyue Liu, Yincang Han, Yonggang Sun, Fajie Gou, Jianyu Chen, Weiqiang Jiang, Qingye Zhao

**Affiliations:** 1 Academy of Animal Husbandry and Veterinary Science, Qinghai University, Xining 810016, Qinghai, China; 2 Qinghai Key Laboratory of Plateau Livestock Genetic Resources Protection and Innovative Utilization, Xining 810016, Qinghai, China

## Abstract

This study aimed to investigate the associations between single-nucleotide polymorphisms (SNPs) and haplotypes in the exon 13 and the intronic regions of the yak *IGF1R* gene and growth traits, with the goal of identifying molecular markers related to growth performance. A total of 400 3-year-old female yaks from the Qinghai Plateau were used as the experimental population. DNA sequencing was employed to detect and genotype SNPs in the *IGF1R* gene. Haploview 3.32 software was used for linkage disequilibrium and haplotype analysis, and the associations between different genotypes and haplotype combinations with growth traits, including body weight, body height, body length, chest circumference, and cannon bone circumference, were evaluated. One SNP (g.7383084C>T) was identified in exon 13, and three SNPs (g.7383136G>A, g.7383137C>T and g.7383177G>A) were identified in intron 13. Haplotype and linkage disequilibrium analyses revealed weak linkage among the four loci, and six haplotypes were identified, among which Hap2 was the dominant one. Association analysis of growth traits indicated that g.7383084C>T, g.7383136G>A, g.7383137C>T, and g.7383177G>A loci were significantly or highly significantly associated with body height and body length in plateau yaks. The advantageous genotypes were CC and CT for g.7383084C>T, GA for g.7383136G>A, CT for g.7383137C>T, and GG for g.7383177G>A. Genotype combination analysis showed that all five common diplotypes were significantly associated with some growth traits, with $H_{2}H_{6}$ being the optimal haplotype combination. These results suggest that polymorphisms in the exon 13 and intronic regions of the *IGF1R* gene in Qinghai Plateau yaks can serve as molecular markers. The significant associations with growth traits provide data-based support for their direct application in the genetic improvement and efficient breeding of yaks.

## 1 Introduction

The yak (*Bos grunniens*) is native to the Qinghai–Tibet Plateau and adjacent alpine regions. As the only large domestic animal adapted to extremely high-altitude environments, it is often referred to as the “ship of the plateau” and “all-purpose livestock” (Wang et al., 2024). It can efficiently utilize alpine meadow forage resources, providing local communities with meat, milk, draft power, hides, fuel, and other livelihood products (Joshi et al., 2020). China's yak population exceeds 15 million heads, accounting for over 95 % of the global total (Han and Liang, 2024). In yak production practices, nutritional regulation measures such as supplementary feeding during the cold season can improve production performance to a certain extent. However, the ultimate level of production performance achievable is fundamentally determined by the yak's genetic potential. With the rapid development of molecular genetics and sequencing technologies, molecular markers have been widely adopted. In livestock, genetic variants, including single-nucleotide polymorphisms (SNPs), small insertions and/or deletions (InDel, < 50 bp ), and copy number variations (CNVs), are widespread across the genome and may contribute to phenotypic diversity and economically important traits (Zhang et al., 2024). As the most commonly used method for assessing genetic diversity in animal populations, SNP molecular markers are characterized by a wide distribution, high density, abundant loci, and high genetic stability within the genome (Guo et al., 2024).

The insulin-like growth factor (IGF) family consists of two ligands (IGF-1 and IGF-2), corresponding receptors (IGF1R and IGF2R), and six binding proteins (IGFBP-1-IGFBP-6) (Annunziata et al., 2011). As multifunctional bioactive peptides, IGFs directly participate in the regulation of animal growth and development; reproductive, nutrition, and metabolic processes; and fundamental cellular activities, including cell proliferation, differentiation, and apoptosis. They also mediate the growth-promoting effects of the growth hormone (GH). As a key receptor in the IGF system, IGF1R mediates its core biological functions and is widely expressed across many tissues and cell types. Therefore, genetic variations in this gene may perturb hormone-signaling pathways and thereby influence growth and reproductive traits. The *IGF1R* is expressed in B and T lymphocytes; in thyroid cells and osteoblasts; and in tissues including the liver, brain, stomach, kidney, heart, lung, muscle, and bone (Denley et al., 2005). During embryonic and postnatal development, IGF1R participates in immune regulation, lymphocyte production, and muscle and bone development (DeChiara et al., 1990). IGF1R is a core component of the neuroendocrine growth axis, and its function depends on the synergistic effect of GH and IGF. In addition to altering the encoded amino-acid sequence, variants in *IGF1R* may influence gene function by affecting pre-mRNA processing, transcript abundance, or receptor expression. Variants within exon 13 or its flanking intronic sequences may disrupt exonic or intronic splicing regulatory elements and, when located near exon–intron boundaries, canonical splice-site signals. Such alterations can affect exon recognition or splice-site selection and may consequently lead to aberrant transcripts, including exon skipping, intron retention, or cryptic splice-site activation (Cartegni et al., 2002; Scotti and Swanson, 2016; Abramowicz and Gos, 2018). Although direct functional evidence for variants in this specific *IGF1R* region remain limited, these potential molecular consequences provide a rationale for investigating *IGF1R* polymorphisms in relation to growth-related traits in yak.

Numerous studies report associations between *IGF1R* polymorphisms and economically important traits in livestock. In cattle, copy number variation (CNV) and insertion/deletion (InDel) polymorphism of *IGF1R* are significantly associated with body weight, withers height, chest girth, and body length (Ma et al., 2019; Tang et al., 2021). Another study reported that SNPs in specific *IGF1R* exons are associated with the weaning weight of Angus cattle (Szewczuk et al., 2013). In addition, *IGF1R* CNV is associated with body weight and withers height in Jinnan cattle and with withers height and hip width in Qinchuan cattle; however, no such associations were detected in Nanyang or Xianan cattle, indicating that its genetic effect may vary by breed (Ma et al., 2019). Beyond growth traits, meta-analyses and transcriptomic data indicate that *IGF1R* participates in the regulation of feed efficiency, AMP-activated protein kinase (AMPK)-related pathways, intramuscular fat (IMF) deposition, and fatty-acid metabolism in beef cattle and promotes muscle cell proliferation and differentiation (Liu et al., 2024; Mota et al., 2022). In sheep, *IGF1R* polymorphisms are also associated with growth performance; one study reported significant associations between several SNPs and five growth traits (Ding et al., 2022). In Polish Merino sheep, the *IGF1R* variant c.654G>A has been associated with average daily gain and foreleg weight, as well as meat quality traits including loin eye depth, intramuscular fat content, and water-holding capacity (Grochowska et al., 2021). In pigs, *IGF1R* expression is associated with fecundity. It shows a distinct pattern in high-fecundity sows, with the highest hepatic expression in replacement gilts at 60 and 90 d of age, suggesting that it may be involved in the developmental regulation of reproductive traits (Pierzchała et al., 2012; Zhao et al., 2018). Therefore, this study aimed to screen *IGF1R* SNPs in Qinghai Plateau yaks, identify loci associated with growth traits, and evaluate their effects through association analyses in order to provide a theoretical basis for molecular-marker-assisted breeding of yaks.

## 2 Material and methods

### 2.1 Experiment material

In Bianma Meilongzhang Cooperative, Yeniugou Township, Qilian County, Haibei Tibetan Autonomous Prefecture, Qinghai Province, 400 healthy adult 3-year-old Qinghai Plateau female yaks were randomly selected as the research objects. First, their growth indicators, including body weight, body length (oblique), body height, chest circumference, and cannon bone circumference, were measured; then 10 mL of whole blood was collected from the jugular vein, anticoagulated with ACD solution at a volume ratio of ( $V_{\text{ACD}}:V_{\text{blood}}$ = 1:6 ), and gently oscillated to mix thoroughly; finally, the treated blood samples were stored at - 80 °C for subsequent genomic DNA extraction and analysis.

### 2.2 Genomic DNA extraction and detection

The genomic DNA of yaks was extracted using a blood genomic DNA extraction kit (Sikejie, Shandong). Subsequently, a NanoDrop 2000 spectrophotometer (Thermo Fisher Scientific, USA) was used to determine the concentration and purity of the extracted DNA, and qualified samples were stored at - 20 °C for later use. The concentration of all DNA samples used in this experiment was higher than 50 $\mathrm{ng}\;µL^{-1}$ , and the ${\mathrm{OD}}_{260}/{\mathrm{OD}}_{280}$ ratio was above 1.8.

**Table 1:** Sequence information of primers.

Primer	Primer sequence (5’-3’)	Fragment length (bp)	Annealing temperature ( °C )	Amplification region
*IGF1R*	F:CTCACCCAGGGTAACCTCACG	216	59	Exon 13, Intron 13
	R:ACGAGCAACAGGGAACAGAAA			

### 2.3 Primer design and PCR amplification

Based on the gene sequence of yak *IGF1R* (NC_091637.1) in GenBank, PCR primers were designed using Primer 5.0 software (Table 1) and synthesized by Sangon Biotech (Shanghai) Co., Ltd.

The 25.0 µL PCR reaction system contained the following components: 12.5 µL mix solution (Sikejie, Shandong, containing deoxynucleoside triphosphates (dNTPs) with nucleic acid dye, Taq DNA polymerase, and 10× Buffer), 9.5 µL ${\mathrm{ddH}}_{2}O$ , 1.0 µL each of forward and reverse primers (20 $µ\mathrm{mol}\;L^{-1}$ ), and 1.0 µL DNA. The amplification reaction was carried out according to the following program: pre-denaturation at 95 °C for 3 min ; 35 cycles of pre-denaturation at 95 °C for 30 s , annealing at 59 °C for 30 s , and extension at 72 °C for 30 s ; and final extension at 72 °C for 5 min , followed by storage at 4 °C . Finally, 0.8 % agarose gel electrophoresis was used to detect the amplification results.

### 2.4 SNP detection

PCR products with good amplification quality were entrusted to Sangon Biotech (Shanghai) Co., Ltd. for purification and sequencing. The obtained sequences were aligned using Seqman software to identify the SNP sites therein.

### 2.5 Data statistical analysis

Based on the genotyping data, the genotype and allele frequencies, genetic heterozygosity (He), effective number of alleles (Ne), and polymorphism information content (PIC) of each polymorphic locus were calculated. SPSS 25.0 software was used to perform a chi-square test on the genotype distribution of the *IGF1R* gene locus in the population, and the Hardy–Weinberg equilibrium (HWE) status was determined according to the following criteria: if $X^{2}<X_{0.05}^{2}$ , the locus was considered to be in equilibrium; if $X_{0.05}^{2}\pm X^{2}<X_{0.01}^{2}$ , it was in disequilibrium; if $X_{0.01}^{2}\pm X^{2}$ , it was in significant disequilibrium. Furthermore, Haploview 3.32 software was used to conduct linkage disequilibrium and haplotype analysis on the four SNP loci of the *IGF1R* gene. To evaluate the effect of different genotypes on growth traits, age and environment were included as covariates in the fixed model to analyze its genetic effect.

$$Y_{ij}=\mu +G_{i}+A_{j}+E_{ij}$$

In the above, $Y_{ij}$ denotes individual record phenotypic, μ denotes population mean, $G_{i}$ denotes marker genotype effect, $A_{j}$ denotes age effect, and $E_{ij}$ denotes random error.

**Figure 1:**
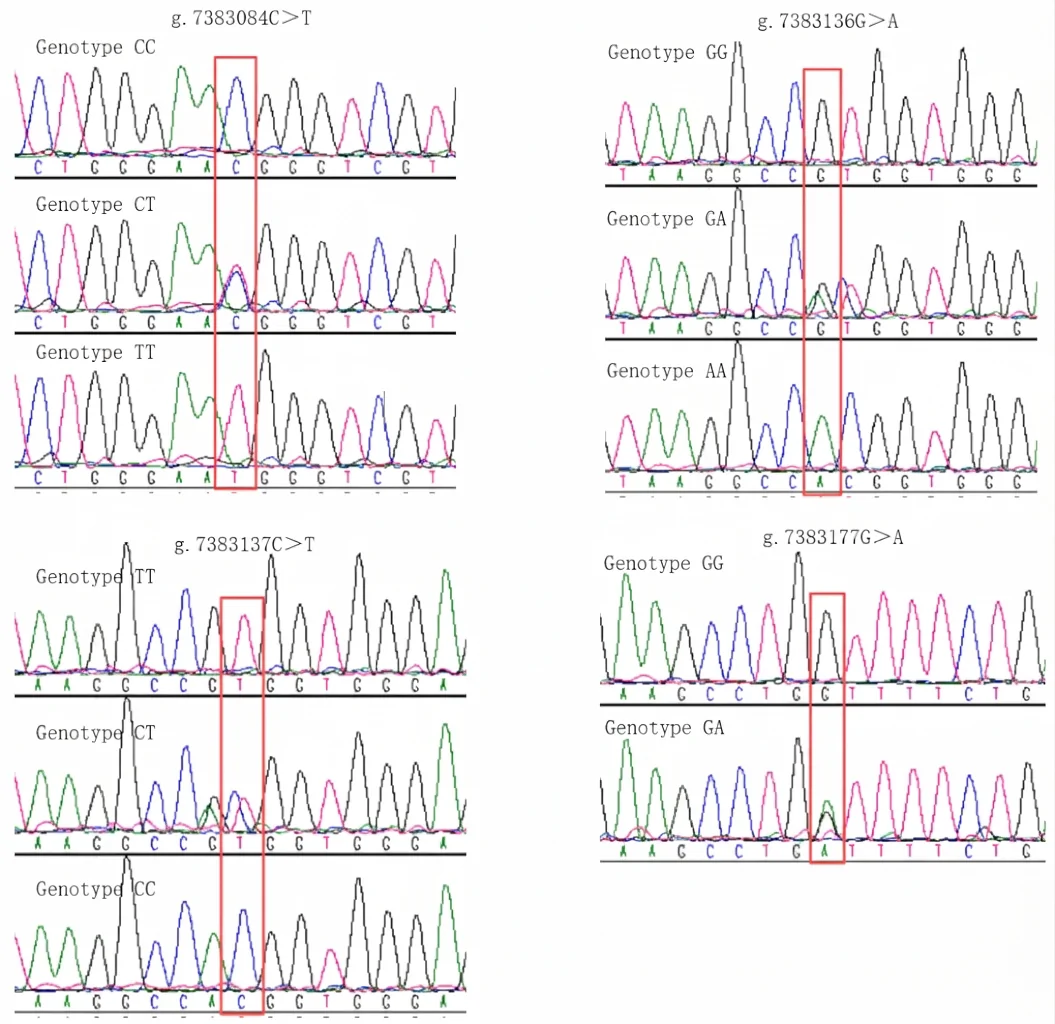
Sequencing of four loci of the *IGF1R* gene. Sanger sequencing chromatograms of different genotypes at four single-nucleotide polymorphism loci. Red boxes indicate the variant sites. The genotypes identified were CC, CT, and TT at g.7383084C>T; GG, GA, and AA at g.7383136G>A; TT, CT, and CC at g.7383137C>T; and GG and GA at g.7383177G>A. Heterozygous genotypes show overlapping peaks corresponding to the two nucleotides, whereas homozygous genotypes show a single peak. Peak colors represent A (green), T (red), C (blue), and G (black).

## 3 Results and analysis

### 3.1 Analysis of genetic characteristics of *IGF1R* gene SNPs

The full length of the *IGF1R* gene in yaks is 305 476 bp, which consists of 21 exons and 20 introns. Sequencing of the amplified *IGF1R* gene fragments in the study population identified four SNPs (Fig. 1). One SNP, g.7383084C>T, was located in exon 13, whereas three SNPs – g.7383136G>A, g.7383137C>T, and g.7383177G>A – were located in intron 13. Both g.7383084C>T and g.7383137C>T have three genotypes, namely CC, CT, and TT; g.7383136G>A has three genotypes, namely GG, GA, and AA; and g.7383177G>A has two genotypes, namely GG and GA.

**Table 2:** Genetic polymorphism analysis of *IGF1R* gene in yak.

Loci	Genotypic frequencies (%)			Allelic frequencies (%)		PIC	He	Ne	HWE test	
									$X^{2}$	P
g.7383084C>T	CC(182)	CT(182)	TT(36)	C	T	0.3395	0.4334	1.7649	0.9948	0.6082
	45.50	45.50	9.00	68.25	31.75					
g.7383136G>A	GG(164)	GA(182)	AA(54)	G	A	0.3554	0.4622	1.8594	0.0967	0.9528
	41.00	45.50	13.50	63.75	36.25					
g.7383137C>T	CC(73)	CT(182)	TT(145)	C	T	0.3668	0.4838	1.9372	1.4175	0.4920
	18.25	45.50	36.25	41.00	59.00					
g.7383177G>A	GG(382)	GA(18)	AA(0)	G	A	0.0430	0.0440	1.0460	0.2119	0.8994
	95.50	4.50	0.00	97.75	2.25					

Note: PIC > 0.5 means high diversity, 0.25 < PIC < 0.5 means moderate diversity, and PIC < 0.25 means low diversity; df = 2, $\chi_{0.05}^{2}$ = 5.991, and $\chi_{0.01}^{2}$ = 9.21; P>0.05 : consistent with HWE.

The results of genetic polymorphism analysis of the yak *IGF1R* gene are shown in Table 2. For the g.7383084C>T site on *IGF1R*, the dominant genotypes are CC and CT, with no single dominant genotype, and the dominant allele is C. The dominant genotype of g.7383136G>A is GA, the dominant genotype of g.7383177G>A is GG, and their dominant alleles are both G. The dominant genotype of the g.7383137C>T mutation site is CT, and the dominant allele is T. The chi-square test showed that all four mutation sites (g.7383084C>T, g.7383136G>A, g.7383137C>T, and g.7383177G>A) are in complete Hardy–Weinberg equilibrium ( P>0.05 ). The g.7383177G>A site is at a low polymorphism level (PIC < 0.25), while g.7383084C>T, g.7383136G>A, and g.7383137C>T are at a moderate polymorphism level (0.25 < PIC < 0.5).

**Table 3:** Effect of different genotypes of *IGF1R* on growth traits in yak.

Loci	Gene type	Body weight	Withers height	Body length	Chest circumference	Cannon bone
		( kg )	( cm )	( cm )	( cm )	circumference ( cm )
g.7383084C>T	CC(182)	137.94 ± 16.61^*a*^	97.54 ± 5.32^*B* *a*^	102.77 ± 7.40^*b*^	133.05 ± 12.27^*B* *b*^	14.87 ± 0.75
	CT(182)	133.57 ± 18.21^*b*^	99.22 ± 6.23^*A*^	104.79 ± 8.87^*a*^	135.83 ± 15.20^*a*^	15.01 ± 0.95
	TT(36)	141.33 ± 16.48^*a*^	96.58 ± 4.56^*b*^	103.50 ± 5.03	139.43 ± 7.69^*A*^	14.99 ± 0.88
g.7383136G>A	GG(164)	137.53 ± 21.56	99.30 ± 6.29^*A*^	105.10 ± 9.01^*A*^	136.10 ± 15.74^*a*^	15.01 ± 0.96
	GA(182)	134.69 ± 15.06	97.51 ± 5.28^*B* *a*^	102.78 ± 7.45^*B*^	133.09 ± 12.33^*b*^	14.87 ± 0.75
	AA(54)	137.72 ± 9.34	97.33 ± 5.17^*b*^	102.93 ± 5.56	137.28 ± 8.27^*a*^	14.99 ± 0.88
g.7383137C>T	CC(73)	130.11 ± 13.19^*B*^	97.85 ± 4.54	103.12 ± 5.70	136.05 ± 9.55	14.76 ± 0.68^*b*^
	CT(182)	138.12 ± 18.40^*A*^	98.78 ± 6.26^*A*^	103.61 ± 8.46^*B*^	135.09 ± 14.15^*a*^	15.02 ± 0.96^*a*^
	TT(145)	137.23 ± 17.31^*A*^	97.69 ± 5.63^*B*^	104.25 ± 8.36^*A*^	134.05 ± 14.35^*b*^	14.94 ± 0.79
g.7383177G>A	GG(382)	136.52 ± 17.78^*a*^	98.31 ± 5.86^*A*^	103.92 ± 8.07^*a*^	135.03 ± 13.62	14.95 ± 0.87
	GA(18)	130.67 ± 8.51^*b*^	96.33 ± 2.47^*B*^	100.22 ± 4.82^*b*^	131.89 ± 10.27	14.83 ± 0.57

Note: data in the table are “mean ± SD”. The difference in terms of lowercase letters in the upper-right corner of the same column indicated significant difference ( P<0.05 ), while the difference in terms of capital letters indicated extremely significant difference ( P<0.01 ); values without superscript letters do not differ significantly from the other genotype groups for the corresponding trait ( P>0.05 ). The same is applied below.

### 3.2 Effects of *IGF1R* gene mutation locus genotype on growth traits in yak

As shown in Table 3, the *IGF1R* g.7383084C>T locus was significantly associated with body weight, withers height, body length, and chest circumference. Carriers of the CC and TT genotypes had significantly higher body weight than carriers of the CT genotype ( P<0.05 ). Withers height was significantly higher in carriers of the CT genotype than in carriers of the CC genotype ( P<0.01 ), whereas carriers of the CC genotype had significantly higher values than carriers of the TT genotype ( P<0.05 ). Carriers of the CT genotype had significantly greater body length than carriers of the CC genotype ( P<0.05 ). For chest circumference, carriers of the TT genotype had significantly higher values than carriers of the CC genotype ( P<0.01 ), whereas carriers of the CT genotype had significantly higher values than carriers of the CC genotype ( P<0.05 ). No significant differences in cannon bone circumference were observed among the genotypes ( P>0.05 ). At the g.7383136G>A locus, the GG genotype had significantly higher withers height and body length than the GA genotype ( P<0.01 ), and the GA genotype had significantly higher withers height than the AA genotype ( P<0.05 ). Chest circumference was significantly higher in carriers of the GG and AA genotypes than in carriers of the GA genotype ( P<0.05 ). No significant differences in body weight or cannon bone circumference were observed among the genotypes ( P>0.05 ). The g.7383137C>T locus was significantly associated with body weight, withers height, body length, chest circumference, and cannon bone circumference. Carriers of the CT and TT genotypes had significantly higher body weight than carriers of the CC genotype ( P<0.01 ). For withers height, carriers of the CT genotype had significantly higher values than carriers of the TT genotype ( P<0.01 ). For body length, carriers of the TT genotype had significantly higher values than carriers of the CT genotype ( P<0.01 ). Chest circumference was significantly higher in carriers of the CT genotype than in those with the TT genotype ( P<0.05 ). Cannon bone circumference was significantly higher in carriers of the CT genotype than in those with the CC genotype ( P<0.05 ). The *IGF1R* g.7383177G>A locus was significantly associated with body weight, body length, and withers height. Carriers of the GG genotype had significantly higher body weight and body length than carriers of the GA genotype ( P<0.05 ). Withers height was also significantly higher in carriers of the GG genotype than in carriers of the GA genotype ( P<0.01 ). No significant differences in chest circumference or cannon bone circumference were observed between the two genotypes ( P>0.05 ).

**Table 4:** Haplotype analysis of *IGF1R* gene mutation site in yak.

Haplotype	g.7383084C>T	g.7383136G>A	g.7383137C>T	g.7383177G>A	Frequency
Hap1	C	A	C	G	0.234
Hap2	C	G	C	G	0.263
Hap3	C	G	T	G	0.160
Hap4	T	A	C	G	0.033
Hap5	T	A	T	G	0.073
Hap6	T	G	T	G	0.199

### 3.3 Haplotype and linkage disequilibrium analysis of *IGF1R* gene SNPs

Haplotype analysis of the four SNPs on the *IGF1R* gene, after excluding haplotypes with a frequency below 3.00 %, detected six haplotypes designated Hap1–Hap6 (Table 4). Haplotype Hap2 had the highest frequency (0.263) and was the major haplotype, while haplotype Hap4 had the lowest frequency (0.033).

**Table 5:** Analysis of *IGF1R* mutation site linkage disequilibrium in yak.

2Loci	g.7383136G>A		g.7383137C>T		g.7383177G>A	
	$D^{\prime }$	$r^{2}$	$D^{\prime }$	$r^{2}$	$D^{\prime }$	$r^{2}$
g.7383084C>T	0.039	0.000	0.750	0.315	0.111	0.001
g.7383136G>A			0.384	0.070	0.233	0.002
g.7383137C>T					0.034	0.000

Linkage disequilibrium analysis revealed (Table 5) no strong linkage among the four SNP loci. The $D^{\prime }$ values between each pair of the four SNPs were all < 0.8, and the $r^{2}$ values were all < 0.33 (in this study, strong linkage disequilibrium was operationally defined as $D^{\prime }$ > 0.8 together with $r^{2}$ > 0.33) (Slatkin, 2008).

**Table 6:** Correlation analysis of *IGF1R* mutation site diplotypes and growth traits in yak.

Double type (quantity)	Body weight ( kg )	Withers height ( cm )	Body length ( cm )	Chest circumference ( cm )	Circumference ( cm )
$H_{2}H_{1}$ (112)	136.56 ± 12.95	98.21 ± 5.00^*b*^	104.57 ± 11.08^*a*^	135.08 ± 10.31^*A*^	14.81 ± 1.23
$H_{2}H_{6}$ (88)	138.06 ± 22.05^*a*^	100.11 ± 6.20^*A* *a*^	105.74 ± 8.50^*A*^	137.64 ± 12.15^*A*^	15.05 ± 0.92
$H_{3}H_{1}$ (61)	131.32 ± 17.93^*b*^	96.48 ± 5.84^*B*^	100.90 ± 7.74^*B* *b*^	129.16 ± 14.64^*B* *b*^	14.81 ± 0.67
$H_{3}H_{6}$ (67)	136.12 ± 25.20	98.28 ± 6.58	104.66 ± 9.64^*a*^	134.55 ± 19.77^*a*^	14.98 ± 1.06
$H_{4}H_{5}$ (25)	139.46 ± 9.59	96.68 ± 4.88^*B*^	103.56 ± 5.16	138.84 ± 8.37^*A*^	15.02 ± 0.96

### 3.4 Effects of combined haplotypes of *IGF1R* gene SNPs on growth traits in yak

As shown in Table 6, combined-haplotype $H_{2}H_{6}$ had significantly higher body weight than $H_{3}H_{1}$ ( P<0.05 ) and significantly higher withers height and body length than $H_{3}H_{1}$ ( P<0.01 ). For withers height, $H_{2}H_{6}$ was significantly higher than $H_{3}H_{1}$ and $H_{4}H_{5}$ ( P<0.01 ) and significantly higher than $H_{2}H_{1}$ ( P<0.05 ). For body length, $H_{3}H_{6}$ and $H_{2}H_{1}$ were also significantly higher than $H_{3}H_{1}$ ( P<0.05 ). Regarding chest circumference, $H_{2}H_{1}$ , $H_{2}H_{6}$ , and $H_{4}H_{5}$ were significantly higher than $H_{3}H_{1}$ ( P<0.01 ), and $H_{3}H_{6}$ was significantly higher than $H_{3}H_{1}$ ( P<0.05 ). Therefore, in this experimental population, the combined haplotype $H_{2}H_{6}$ may be the optimal combined haplotype influencing growth traits in yaks.

## 4 Discussion

Growth traits are key indicators of livestock growth, development, and economic value, playing a significant role in breeding. Animal growth and development are precisely regulated by the growth hormone GH–IGF1 axis, which plays a crucial role in body growth, development, and anabolic processes. As the core regulator of this pathway, GH biological functions are primarily mediated through the binding of IGF1 to its receptor, *IGF1R* (Renaville et al., 2002). *IGF1R* has a high affinity for IGF1 and is considered to be a potential quantitative trait locus (QTL) that influences traits like growth, carcass traits, and reproductive performance (Kawashima et al., 2005). In the present study, several favorable genotypes of *IGF1R* were associated with yak body measurement traits. Specifically, the favorable genotypes were CC and CT at g.7383084C>T, GA at g.7383136G>A, CT at g.7383137C>T, and GG at g.7383177G>A. Individuals carrying these genotypes showed significant differences in withers height, body length, and related body measurement traits compared with individuals carrying other genotypes. This observation is consistent with previous reports in multiple Chinese yak populations, including Datong yak, Qinghai Plateau yak, Tianzhu White yak, Gannan yak, and Xinjiang yak, in which polymorphism variants located in exon 1 of the *IGF1R* gene were shown to exert significant effects on early postnatal growth and development (Liang et al., 2010). Combined with these earlier findings, our results further support the hypothesis that sequence polymorphisms in different regions of the yak *IGF1R* gene, both in exons and in flanking introns, can contribute to phenotypic variation in growth traits across different developmental stages and genetic backgrounds. These marker trait associations are biologically plausible because *IGF1R* is a key upstream mediator of IGF1-dependent signaling that directly regulates chondrocyte proliferation, longitudinal bone growth, and systemic skeletal development, all of which are fundamental processes underlying body size and body conformation formation in yaks (Kawashima et al., 2005). Therefore, the variants identified in the exon 13–intron 13 region of the yak *IGF1R* gene may be considered to be promising candidates for marker-assisted selection aiming at improving growth performance in local yak-breeding programs.

The effective allele number, heterozygosity, and polymorphic information content are key indicators of a gene fragment’s polymorphism and the population’s genetic variation. These indicators reflect genetic similarity among individuals within a population; higher values generally indicate greater genetic variation. In family-based breeding systems, genetic loci with a large effective number of alleles, high polymorphism information content, and high heterozygosity can be used for marker-assisted selection in conjunction with association analyses of important production traits, thereby providing a genetic basis for improving livestock and poultry production performance. In the present study, four SNPs were detected in the exon 13–intron 13 region of the yak *IGF1R* gene, including g.7383084C>T, g.7383136G>A, g.7383137C>T, and g.7383177G>A. At the g.7383084C>T locus, 182 individuals had the CC genotype, 182 had the CT genotype, and 36 had the TT genotype. At the g.7383136G>A locus, 164, 182, and 54 individuals carried the GG, GA, and AA genotypes, respectively. At the g.7383137C>T locus, the numbers of individuals with the CC, CT, and TT genotypes were 73, 182, and 145, respectively. At the g.7383177G>A locus, 382 individuals carried the GG genotype, whereas 18 carried the GA genotype. The exonic variant g.7383084C>T was a synonymous substitution that did not alter the encoded amino acid as both alleles encoded aspartic acid. However, synonymous variants should not be regarded as functionally neutral a priori because they may influence codon usage bias, mRNA secondary structure, mRNA stability, translation efficiency, co-translational protein folding, or exonic-splicing regulatory elements (Sauna and Kimchi-Sarfaty, 2011; Hunt et al., 2014; Wang et al., 2020). Therefore, g.7383084C>T may affect yak growth traits by modulating *IGF1R* transcript processing or expression efficiency, or it may be in linkage disequilibrium with another functional variant in the *IGF1R* gene. The variants g.7383136G>A and g.7383137C>T were located 9 and 10 bp downstream of exon 13, respectively, suggesting that they are near-exon intronic variants positioned close to the exon–intron boundary, whereas g.7383177G>A was located within intron 13. Although intronic variants do not alter the amino acid sequence directly, variants close to exon–intron boundaries may affect pre-mRNA splicing by altering splice donor recognition, exon definition, intronic splicing enhancers, intronic splicing silencers, or other cis-regulatory elements (Chiang et al., 2022). In particular, intronic positions near + 9 and + 10 downstream of an exon may lie within regions relevant to splice-site recognition and early spliceosomal assembly, suggesting that g.7383136G>A and g.7383137C>T could potentially influence *IGF1R* transcript structure, transcript abundance, or isoform composition (Pagani and Baralle, 2004; Chiang et al., 2022). The intronic variant g.7383177G>A is farther from the exon–intron boundary and exhibited low polymorphism (PIC < 0.25), indicating limited genetic variability and relatively weak potential for single-locus selection; nevertheless, it may still contribute to phenotypic variation as part of a haplotype background. All four loci conformed to Hardy–Weinberg equilibrium ( P>0.05 ), suggesting that they were in a relatively stable genetic state in the sampled yak population. By contrast, g.7383084C>T, g.7383136G>A, and g.7383137C>T showed moderate polymorphism (0.25 < PIC < 0.5), indicating that these loci retain sufficient allelic diversity for evaluating genotype–phenotype associations and may serve as useful candidate markers for marker-assisted selection of yak growth traits. Taken together, the synonymous exonic variant and near-exon intronic variants identified in this study may influence yak growth traits through regulatory mechanisms affecting *IGF1R* mRNA processing, expression, or linkage with causal variants.

From a quantitative genetic perspective, additive genetic effects represent an important component of heritable variation in complex traits and provide the basis for allele substitution effects and breeding-value prediction (Falconer and Mackay, 1996; Lynch and Walsh, 1998; Mrode, 2014). For a biallelic locus, a potential additive pattern is suggested when phenotypic values change progressively with the number of alternative alleles and the heterozygous genotype is approximately intermediate between the two homozygous genotypes. In the present study, formal additive–dominance models were not fitted; therefore, the observed genotype differences cannot be regarded as direct evidence of additive genetic effects. Nevertheless, some genotype means showed potential allele dose tendencies for individual traits. For example, chest circumference at g.7383084C>T increased from CC to CT and TT, suggesting a possible positive dose-related association of the T allele with chest circumference. At g.7383137C>T, body length showed an increasing tendency from CC to CT and TT, whereas chest circumference showed a decreasing tendency with increasing T-allele dosage. These trait-specific patterns suggest that some *IGF1R* variants may contribute to phenotypic variation through potentially additive or partially additive mechanisms. However, several traits did not follow a strictly linear genotype–phenotype pattern, and heterozygote superiority or genotype-specific effects were observed at some loci. Such patterns may reflect dominance, overdominance, linkage disequilibrium with functional variants, or differences in local haplotype background. Therefore, the present findings should be interpreted as preliminary evidence of potential allele dose tendencies (Falconer and Mackay, 1996; Lynch and Walsh, 1998; Mackay et al., 2009). In addition to single-locus effects, the joint inheritance of linked variants, as captured by haplotypes and diplotypes, may also contribute to phenotypic variation in animal growth traits. Previous studies have reported associations between *ADIPOQ* haplotype combinations and body measurements in Qinchuan cattle (Zhang et al., 2013), as well as associations between *Dapper1* haplotypes and body weight across several cattle breeds (Wang et al., 2013). Consistently with these observations, all five *IGF1R* diplotype combinations in the present study showed significant or highly significant associations with several growth traits. Comprehensive comparison of the measured traits suggested that the $H_{2}H_{6}$ diplotype was associated with relatively favorable growth performance in the sampled yak population.

## 5 Conclusions

The *IGF1R* gene polymorphism in yaks and all four identified polymorphic sites are all significantly associated with growth traits, with H2H6 being the superior diplotype. Therefore, *IGF1R* can be considered to be a candidate gene for marker-assisted selection in yaks. Incorporating these markers into breeding programs is expected to enhance selection efficiency, shorten the breeding cycle, and promote sustainable development of the yak industry.

## Data Availability

The datasets generated and analyzed during the current study are available from the corresponding author on reasonable request.
